# Antimicrobial Resistance Characterization of Methicillin-Resistant *Staphylococcus aureus* and *Staphylococcus pseudintermedius* Isolates from Clinical Cases in Dogs and Cats in Belgium

**DOI:** 10.3390/antibiotics14070631

**Published:** 2025-06-20

**Authors:** Suzanne Dewulf, Filip Boyen, Dominique Paepe, Cécile Clercx, Noah Tilman, Jeroen Dewulf, Cécile Boland

**Affiliations:** 1 Veterinary Epidemiology Unit, Ghent University, 9820 Merelbeke, Belgium; suzanne.dewulf@ugent.be (S.D.); jeroen.dewulf@ugent.be (J.D.); 2 Veterinary Bacteriology Service, Sciensano, 1050 Brussels, Belgium; noah.tilman@sciensano.be; 3 Department of Pathobiology, Pharmacology and Zoological Medicine, Faculty of Veterinary Medicine, Ghent University, Salisburylaan 133, 9820 Merelbeke, Belgium; filip.boyen@ugent.be; 4 Department of Small Animal Medicine and Clinical Biology, Ghent University, 9820 Merelbeke, Belgium; dominique.paepe@ugent.be; 5 Department of Companion Animal Clinical Sciences, University of Liège, 4000 Liège, Belgium; cclercx@ulg.ac.be

**Keywords:** MRSA, MRSP, *mecA*, *mecC*, multidrug-resistant organisms, dogs, cats, clinical, resistance

## Abstract

**Background/Objectives**: Methicillin-resistant *Staphylococcus aureus* (MRSA) and methicillin-resistant *Staphylococcus pseudintermedius* (MRSP) represent important antimicrobial resistance threats related to companion animals, which can directly or indirectly lead to adverse health effects in humans and animals living in close contact. Characterizing the phenotypic resistance of MRSA and MRSP to a panel of antimicrobials relevant to both veterinary and human medicine is crucial within a “One Health” framework. **Methods**: In this study, a total of 79 presumptive MRSA isolates (34 from cats, 45 from dogs) and 110 presumptive MRSP isolates (105 from dogs, 5 from cats) from clinical cases were analysed. Real-time PCR was used to detect the presence of *mecA* and *mecC* genes, and susceptibility testing was performed using the Sensititre EUST2 panel. **Results**: Most of the isolates (88.9%, 168/189) were positive for the *mecA* gene, while a minority (1.1%, 2/189) were *mecC*-positive (2 MRSA, 1 dog, 1 cat). MRSP isolates exhibited acquired resistance to a broader range of antibiotics compared to MRSA strains. Furthermore, several isolates demonstrated acquired resistance to antibiotics considered critically important for human medicine. Resistance to vancomycin was found in an MRSP isolate from a dog, and resistance to linezolid in an MRSP isolate from a cat. This study reveals that 83.3% (30/36) of MRSA isolates from dogs and 89.3% (25/28) from cats were multidrug-resistant organisms, while MRSP isolates exhibited multidrug resistance in 99% (101/102) of cases for dogs and 100% (4/4) for cats. **Conclusions**: The extremely high level of multidrug resistance, with some isolates resistant to critically important antibiotics used in human medicine, highlight the importance of monitoring antimicrobial susceptibility in MRSA and MRSP isolates collected from cats and dogs in a One Health perspective.

## 1. Introduction

*Staphylococcus aureus* and *Staphylococcus pseudintermedius* are both Gram-positive, coagulase-positive bacteria. *S. aureus* is a commensal organism and opportunistic pathogen commonly found in the nasal flora of humans, but it also colonizes various animals, including dogs, cats, horses, pigs, rabbits, poultry and cattle [1,2,3]. *S. pseudintermedius*, on the other hand, is primarily a commensal bacterium found on the skin and mucous membranes of dogs and cats [4], though some studies suggest that *S. pseudintermedius* might not be part of the natural microbiota of cats [5,6]. Both species can exploit disruptions in the skin or mucosa to invade adjacent tissues or enter the bloodstream, potentially leading to infection. This is often observed in cases involving chronic dermatological conditions, post-surgical infections, or immune suppression. *S. aureus* is recognized as one of the most clinically significant bacteria responsible for nosocomial infections in humans, dogs, and cats [3,7]. Meanwhile, *S. pseudintermedius* is the leading cause of skin, ear, soft tissue, and cavity infections in dogs [8], and it occasionally causes similar infections in cats [9]. Both bacteria have a zoonotic potential, and given the close interactions between humans and animals, there is a significant risk of exchanging resistant bacteria or resistance genes, which poses public health challenges [10,11]. In recent years, the evolving social status of pets, now seen as family members, has led to more frequent and intense interactions between pets and their owners [10]. This shift has significant implications for public health due to the zoonotic potential of antimicrobial-resistant pathogens. Several studies have demonstrated that methicillin-resistant *Staphylococcus aureus* (MRSA) strains isolated from companion animals are typically of human origin, indicating reverse zoonosis, a transmission of pathogens from humans to animals [12,13]. These host-switching events —where pathogens are transmitted and adapted between humans and animals—pose risks to public health, animal health, and welfare, as they facilitate the spread of resistant strains such as MRSA and MRSP [14]. Notably, outbreaks of methicillin-resistant *Staphylococcus pseudintermedius* (MRSP) have been reported in veterinary clinics [4,15], particularly affecting dogs and their owners, highlighting the role of the veterinary environment as a potential reservoir for multidrug-resistant organisms [16,17].

To address these challenges, antimicrobial stewardship is critical. The widespread use of penicillin and its derivatives (e.g., ampicillin, amoxicillin) in both human and veterinary medicine has contributed to the emergence of resistant strains, such as MRSA and MRSP. These strains are characterized by acquired resistance to most beta-lactam antibiotics, including penicillin, amoxicillin, and cephalosporins [18]. In wild-type *S. aureus* and *S. pseudintermedius*, beta-lactam antibiotics inhibit cell wall synthesis by binding to penicillin-binding proteins (PBPs), which are critical for cross-linking peptidoglycan chains. This disruption leads to bacterial cell lysis. However, MRSA and MRSP produce an altered penicillin-binding protein, PBP2a, which has a low affinity for beta-lactam antibiotics. Consequently, these bacteria can continue synthesizing their cell walls in the presence of most beta-lactams [19]. The gene encoding PBP2a, *mecA*, is carried on a large mobile genetic element known as the staphylococcal chromosomal cassette (*SCCmec*). In 2011, Garcia Alvarez L. et al. [19] discovered a novel *mecA* homolog, *mecC*, in human and bovine populations in the UK and Denmark. This gene encodes a PBP2a/2′ protein, and its role in beta-lactam resistance was demonstrated in subsequent studies. Notably, PBP2a has a higher affinity for cefoxitin than oxacillin, whereas PBP2a/2′ has a higher affinity for oxacillin than cefoxitin [20]. While *mecC*-MRSA was initially found in dairy cattle and later in humans, studies have also reported its prevalence in hedgehogs in Denmark and Sweden [21]. In various European countries, the presence of *mecC*-MRSA in sheep, horses, and dogs has been described [22,23]. It is noteworthy that *SCCmec* is also a vehicle for resistance genes other than *mecA* and *mecC*, which confer resistance to a broad range of antimicrobials and enhance the bacteria’s adaptability to hostile environments [24]. MRSA and MRSP can thus evolve into multidrug-resistant organisms (MDROs). MDROs are designated as such when they show acquired resistance to at least one agent belonging to at least three different antimicrobial classes [25,26].

Moreover, monitoring resistance to antibiotics classified in category A (“Avoid”)—as defined by the Antimicrobial Advice Ad Hoc Expert Group (AMEG) of the European Medicines Agency (EMA)—is particularly important for protecting human health [27]. This category includes vancomycin, linezolid, rifampicin, mupirocin, and quinupristin/dalfopristin. Some antibiotic classes that are not authorised for veterinary use but are approved for human medicine in the European Union may be used, on an exceptional basis, in non-food-producing animals, in compliance with the prescribing “cascade”. More precisely, as outlined in Articles 10 and 11 of Directive 2001/82/EC and Articles 107, 112, 113, and 114 of Regulation (EU) 2019/6, the legislation provides a framework that allows veterinarians—when no suitable authorised product is available and in exceptional cases—to either use a veterinary medicinal product outside its authorised conditions or to prescribe an unauthorised medicine, following specific criteria defined by the so-called cascade system. Individual substances that are not authorised for use in veterinary medicine but are in antibiotic (sub)classes included in Categories B, C or D (e.g., azithromycin, which is in the macrolide class) may also only be administered under the “cascade”. The diagnosis of MRSA or MRSP in an animal should be treated with the most appropriate antibiotic, in accordance with the results of the antibiogram.

This study explored for the first time, to our knowledge, a collection of MRSA and MRSP strains isolated from clinical cases in cats and dogs in Belgium. This research aimed to characterize the phenotypical resistance to several classes of antibiotics relevant to both veterinary and human medicine and the presence of either the *mecA* or *mecC* gene in this collection, to gather missing data on these zoonotic bacteria to strengthen the European One Health antimicrobial resistance surveillance approach, as promoted by the EARS-Vet network [14].

## 2. Results

A total of 79 presumptive MRSA isolates (45 from dogs and 34 from cats) and 110 presumptive MRSP isolates (105 from dogs and 5 from cats) were received and analysed using RT-PCR and antimicrobial susceptibility testing. MRSA isolates were confirmed when both the *nuc* gene (confirming identification as *Staphylococcus aureus*) and either the *mecA* or *mecC* gene were detected. This resulted in 64 confirmed MRSA (36 dogs, 28 cats) and 106 confirmed MRSP (102 dogs, 4 cats) isolates from clinical cases in dogs and cats.

### 2.1. MRSA

**Figure 1:**
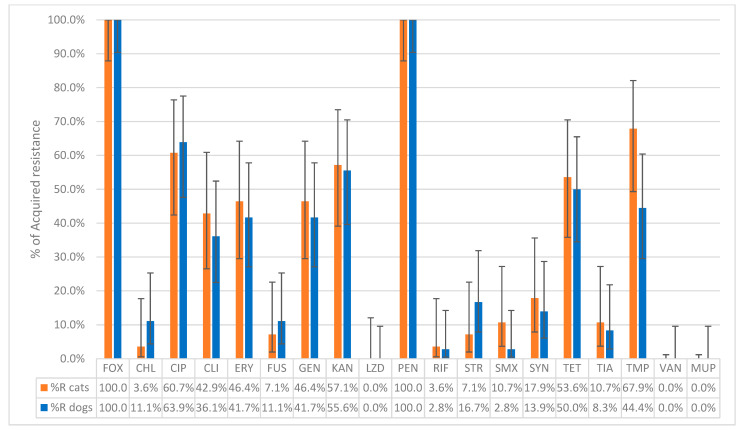
The percentage of non-wild-type isolates of MRSA collected from 36 dogs and 28 cats to the tested antibiotics. FOX: Cefoxitin; CHL: Chloramphenicol; CIP: Ciprofloxacin; CLI: Clindamycin; ERY: Erythromycin; FUS: Fusidic acid; GEN: Gentamicin; KAN: Kanamycin; LZD: Linezolid; PEN: Penicillin; RIF: Rifampicin; STR: Streptomycin; SMX: Sulfamethoxazole; SYN: Quinupristin–Dalfopristin; TET: Tetracycline; TIA: Tiamulin; TMP: Trimethoprim; VAN: Vancomycin; MUP: Mupirocin, Sensititre^®^ EUST2 panel.

Among the *nuc*-positive isolates, 79.5% (35 dogs, 27 cats) carried the *mecA* gene, 2.6% (1 dog, 1 cat) carried the *mecC* gene, while in 17.9% (8 dogs, 6 cat), neither of the *mec* genes was detected. Only the strains that possessed the *nuc* and *mecA* or *mecC* genes (36 dogs and 28 cats) were considered as confirmed MRSA isolates and were used for further susceptibility testing. The results were interpreted according to the most recent ECOFFs (Table 1). The susceptibility testing of MRSA from dogs confirmed that 100% (36/36) of the isolates exhibited acquired resistance to cefoxitin and penicillin. Acquired resistance was also observed in ciprofloxacin (63.9%, 23/36), kanamycin (55.6%, 20/36), tetracycline (50.0%, 18/36), trimethoprim (44.4%, 16/36), erythromycin and gentamicin (both 41.7%, 15/36), and clindamycin (36.1%, 13/36). Lower percentages were found for streptomycin (16.7%, 6/36), chloramphenicol and fusidic acid (both 11.1%, 4/36), tiamulin (8.3%, 3/36), and sulfamethoxazole (2.8%, 1/36). No acquired resistance to linezolid, vancomycin, or mupirocin was observed. As for antibiotics belonging to the AMEG category A, resistance was detected in 13.9% (5/36) of isolates for quinupristin–dalfopristin and in 2.8% (1/36) for rifampicin (Figure 1).

Among MRSA isolates from cats, 100% (28/28) showed acquired resistance to cefoxitin and penicillin. Resistance was also observed in trimethoprim (67.9%, 19/28), ciprofloxacin (60.7%, 17/28), kanamycin (57.1%, 16/28), tetracycline (53.6%, 15/28), erythromycin and gentamicin (both 46.4%, 13/28), and clindamycin (42.9%, 12/28). Lower percentages were found for tiamulin and sulfamethoxazole (both 10.7%, 3/28), fusidic acid and streptomycin (both 7.1%, 2/28), and chloramphenicol (3.6%, 1/28). No acquired resistance to linezolid, vancomycin, or mupirocin was observed. As for the antibiotics belonging to the AMEG category A, acquired resistance was observed in quinupristin–dalfopristin (17.9%, 5/28) and in rifampicin (3.6%, 1/28) (Figure 1). The distribution of the MIC values of the 19 tested antimicrobials for the 64 MRSA isolates is shown in Table 2. Chi-square tests performed to compare the proportions of MRSA isolates with acquired resistance between dogs and cats for each antibiotic revealed no statistically significant differences (all *p*-values > 0.05).

**Figure 2:**
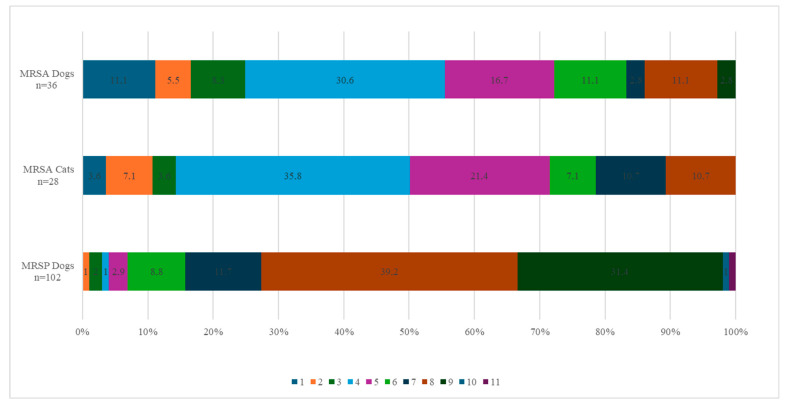
Percentage of MRSA and MRSP strains from dogs and cats resistant to the corresponding number of antibiotic families. The colour legend indicates the number of families of antibiotics to which the strains are resistant. The abscissa indicates the percentage of strains resistant to the corresponding number of antibiotic families.

Out of the 36 MRSA isolates from dogs, 83.3% (30/36) were considered MDROs, with 30.6% (11/36) of the isolates exhibiting acquired resistance to four classes of antibiotics and 2.8% (1/36) exhibiting acquired resistance to a maximum of nine different classes. Among the cat MRSA isolates, 89.3% (25/28) were MDROs, with 35.7% (10/28) exhibiting acquired resistance to four antibiotic classes and 10.7% (3/28) exhibiting acquired resistance to a maximum of eight antibiotic classes (Figure 2). The resistance profiles for the 36 dogs and 28 cats can be found in Table S1.

### 2.2. MRSP

**Figure 3:**
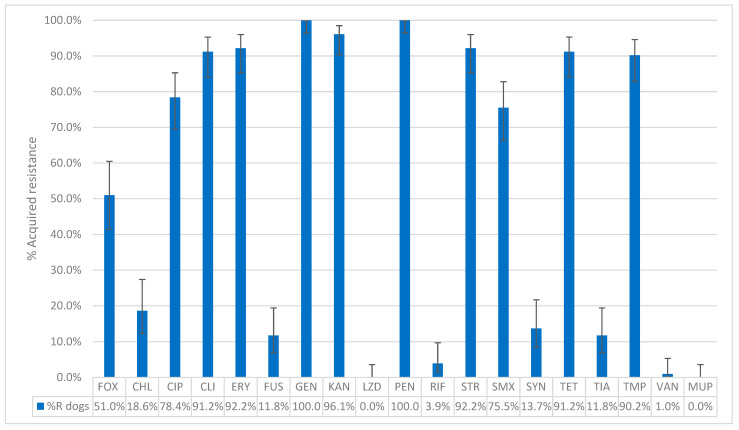
The percentage of acquired resistance in non-wild type isolates of MRSP collected from 102 dogs to the tested antibiotics. FOX: Cefoxitin; CHL: Chloramphenicol; CIP: Ciprofloxacin; CLI: Clindamycin; ERY: Erythromycin; FUS: Fusidic acid; GEN: Gentamicin; KAN: Kanamycin; LZD: Linezolid; PEN: Penicillin; RIF: Rifampicin; STR: Streptomycin; SMX: Sulfamethoxazole; SYN: Quinupristin–Dalfopristin; TET: Tetracycline; TIA: Tiamulin; TMP: Trimethoprim; VAN: Vancomycin; MUP: Mupirocin. Sensititre^®^ EUST2 panel.

Among the 110 presumptive MRSP isolates (105 dogs, 5 cats), 96.4% (102 dogs, 4 cats) carried the *mecA* gene and were therefore confirmed as MRSP. Four isolates (3 dogs, 1 cat) tested negative for both *mecA* and *mecC* genes and were not retained as confirmed MRSP. This resulted in 102 confirmed MRSP isolates from dogs and 4 from cats. In dogs, all MRSP isolates (100%, 102/102) exhibited acquired resistance to penicillin, while 51.0% (52/102) showed acquired resistance to cefoxitin. Acquired resistance was also observed in gentamicin (100%, 102/102), kanamycin (96.1%, 98/102), erythromycin and streptomycin (both 92.2%, 94/102), clindamycin and tetracycline (both 91.2%, 93/102), trimethoprim (90.2%, 92/102), ciprofloxacin (78.4%, 80/102), and sulfamethoxazole (75.5%, 77/102). Lower percentages were found for chloramphenicol (18.6%, 19/102), fusidic acid, and tiamulin (both 11.8%, 12/102). Regarding antimicrobials belonging to the AMEG category A, 13.7% (14/102) of the isolates showed acquired resistance to quinupristin–dalfopristin, 3.9% (4/102) to rifampicin, and 1.0% (1/102) to vancomycin. All MRSP isolates from dogs belonged to the wild-type population for linezolid and mupirocin (Figure 3). The distribution of the MIC values for the 19 tested antimicrobials is shown in Table 3. Among the dog isolates, 99% (101/102) were MDROs, with 39.2% (40/102) exhibiting acquired resistance to 8 classes of antimicrobial agents and one isolate (1.0%) showing acquired resistance to 11 antimicrobial classes (Figure 2).

Among the four MRSP isolates from cats, all isolates (4/4) showed acquired resistance to penicillin, ciprofloxacin, erythromycin, gentamicin, kanamycin, and trimethoprim. Additionally, three isolates (3/4) displayed acquired resistance levels for clindamycin, streptomycin, sulfamethoxazole and tetracycline; two isolates (2/4) to cefoxitin and one (1/4) to tiamuline. Regarding AMEG category A antimicrobials, one isolate (1/4) exhibited acquired resistance to both quinupristin–dalfopristin and linezolid and another isolate (1/4) to rifampicin The four isolates from cats were MDROs, with a minimum of acquired resistance to 5 antimicrobial classes and a maximum of acquired resistance to 11 antimicrobial classes (Table 4). Given the large discrepancy in the number of MRSP isolates from dogs (n = 102) compared to cats (n = 4), statistical analyses to compare resistance patterns between the two species were not conducted. The resistance profiles for the 102 dogs and 4 cats can be found in Table S2.

## 3. Discussion

This study aimed to characterize the antimicrobial resistance patterns of 106 confirmed MRSP and 64 confirmed MRSA isolates from clinical cases of dogs and cats. Specifically, it focused on assessing the prevalence of acquired resistance to 19 antimicrobials in canine and feline isolates and evaluating the occurrence of MDROs as well as detecting methicillin-resistance genes.

Most of the presumptive MRSA isolates (78.5%) carried the *mecA* gene, with a small percentage (2.5%) carrying *mecC*. In contrast, nearly all presumptive MRSP strains (96.4%) were *mecA*-positive, with none carrying *mecC*. Thus, 14 presumptive MRSA and 4 presumptive MRSP did not carry the *mecA* or the *mecC* gene, while they were initially reported as methicillin-resistant based on susceptibility testing in routine diagnostics. Methicillin resistance in *mecA*- or *mecC*-negative isolates can still occur, mediated by alternative mechanisms. One such mechanism is the production of beta-lactamase, an enzyme capable of breaking down beta-lactam antibiotics and rendering them ineffective [29]. This enzyme is encoded by *blaZ*, which resides on a large transposon on a plasmid. Another mechanism involves mutations in native penicillin-binding proteins (PBPs), which alter their structure, reducing their binding affinity to beta-lactams and thereby conferring resistance without the involvement of PBP2a [30]. In these rare cases, methicillin resistance may still be observed phenotypically, even if molecular tests detecting *mecA*/*mecC* detection are negative. However, without the presence of *mecA* or *mecC*, these isolates would typically not be classified as MRSA or MRSP, since these genes are indicators for methicillin resistance in these organisms [31,32].

All confirmed MRSA isolates from both dogs and cats exhibited acquired resistance to cefoxitin and penicillin. Consistently, all confirmed MRSP isolates showed acquired resistance to penicillin. However, only 51% of confirmed MRSP isolates exhibited phenotypic resistance to cefoxitin. This lower level of acquired resistance to cefoxitin in MRSP (51%) compared to MRSA (100%) is not surprising. Several studies have demonstrated that cefoxitin can lead to false-negative results when used to detect methicillin resistance in MRSP [4,33,34]. This is because cefoxitin does not adequately induce *mecA* expression in *S. pseudintermedius*, leading to under-detection of resistant isolates. For *S. pseudintermedius*, oxacillin susceptibility testing is recommended as a more reliable indicator of methicillin resistance. The Clinical Laboratory Standards Institute (CLSI) has published guidelines suggesting the use of oxacillin rather than cefoxitin for *S. pseudintermedius* to improve sensitivity and avoid false-negative results [31,35]. Cefoxitin, while useful for *S. aureus*, has been shown to have poor sensitivity in *S. pseudintermedius*, with studies reporting sensitivities as low as 6.7% for *mecA*-positive MRSP isolates [32]. The primary aim of the present study was not to assess the susceptibility of MRSP isolates to cefoxitin, nor to estimate the prevalence of MRSP in pets, but to evaluate their susceptibility to a panel of antimicrobials from a ready-to-use plate originally designed for the monitoring of MRSA. Except for cefoxitin, all other antimicrobials targeted by this plate fit the purpose. Our side results observed for cefoxitin confirmed observations from previous studies.

This study also revealed two *mecC*-positive MRSA isolates (one dog and one cat). To our knowledge, this is the first report of *mecC* detection in dogs and cats in Belgium; however, *mecC*-positive MRSA has previously been identified in rats in the country [36]. But other cases have already been detected in animals in Austria, Brazil, and Portugal [37,38], and one *mecC* isolate has been found in a cat in Norway [39]. It is noteworthy that the *mecC* gene was never observed in food-producing animals in Belgium across the national monitoring conducted since 2011. These findings highlight that different MRSA clones are found in companion animals compared to food-producing animals.

Regarding the antimicrobial susceptibility to other antimicrobials, very high levels of acquired resistance were observed for ciprofloxacin, kanamycin, trimethoprim and tetracycline in feline MRSA isolates, while canine isolates displayed very high levels of acquired resistance to ciprofloxacin and kanamycin. For MRSP isolates, extremely high levels of acquired resistance to gentamicin, kanamycin, erythromycin, streptomycin, clindamycin, tetracyclines, trimethoprim, ciprofloxacin, and sulfamethoxazole were found in dog isolates. In cats, all the four MRSP isolates showed acquired resistance to multiple antimicrobials, including ciprofloxacin, erythromycin, gentamicin, kanamycin, and trimethoprim. Particular attention must be paid when interpreting MRSP isolates using the ECOFFs established for MRSA (CHL, CIP, FOX, FUS, LZD, RIF, STR, SYN, TIA, TMP, VAN, and MUP), tentative ECOFFs (KAN), or approximation (SMX) (Table 1). Certain strains with MIC values close to the threshold may still be wild-type. It is important to note that, with the establishment of ECOFFs for MRSP in the future, antimicrobial resistance surveillance will become more robust and better suited to its purpose.

Compared to MRSA, MRSP isolates demonstrated a higher level of multidrug resistance to a broader range of antimicrobials, with 99% in dogs and 100% in cats (n = 4). In contrast, 83.3% of MRSA isolates from dogs and 89.3% from cats were MDROs. Other studies also highlighted that MRSP isolates often display acquired resistance to a broader range of antimicrobial classes, including aminoglycosides, macrolides, lincosamides, tetracyclines, and fluoroquinolones [40]. This is due to the presence of diverse *SCCmec* elements and additional resistance, which have contributed to MRSP’s multidrug resistance profile, making it more resistant than MRSA. Also, MRSP might be exposed to different selective pressures than MRSA. A study highlighted that the use of beta-lactams in veterinary practice may be less consistent or standardized than in human medicine [41] and there is extensive use of a wider range of antibiotics in veterinary medicine, including classes not commonly used in humans [31]. The study by Joosten et al. (2020) [42] further discusses the widespread use of antimicrobials in companion animals, which has contributed to the emergence of resistant pathogens like MRSP. Similar results were presented in a study conducted with MRSP isolates in dogs from several countries in Europe, the USA and Canada, and another study showed even higher resistance rates to quinolones, aminoglycosides and macrolides in MRSP strains from dogs in Germany [40,43]. Over the two years of sample collection, it was easier to obtain MRSP clinical isolates compared to MRSA. This can be attributed to the fact that MRSA is primarily a human-associated pathogen [44], while MRSP is more specific to pets [4]. Additionally, a greater number of MRSP isolates were collected from dogs than from cats, which is likely due to conditions such as pyoderma and otitis externa, which are frequently caused by MRSP and are more common in dogs than in cats. Moreover, *S. pseudintermedius* is not typically part of the natural microbiota of cats [5]. The presence of MRSP in cats may therefore result from close contact with dogs [45].

This study identified acquired resistance to antimicrobials of the highest importance for humans (i.e., classified in the AMEG category A) in some isolates, including vancomycin, linezolid, rifampicin, and quinupristin–dalfopristin. Specifically, one MRSP isolate (dog) showed acquired resistance to vancomycin, and one cat MRSP isolate exhibited acquired resistance to linezolid, while rifampicin-acquired resistance was observed in two MRSA isolates (one dog, one cat) and five MRSP isolates (four dogs, one cat). Additionally, 10 MRSA (5 dogs, 5 cats) and 14 MRSP isolates (13 dogs, 1 cat) exhibited acquired resistance to quinupristin–dalfopristin, even though these antibiotics are not available for use in veterinary medicine. Linezolid, rifampicin, and vancomycin are considered critically important antimicrobials in human medicine, particularly as last-resort treatments for infections caused by multidrug-resistant organisms [46]. So far, this would be the first linezolid-resistant MRSP (LR-MRSP) described in a cat, and this has never been reported previously in any animal species worldwide, to our knowledge. This should be confirmed in the future, when ECOFFs for MRSP will be available for linezolid. While linezolid-resistant strains in pets are still rare, the identification of LR-MRSP in companion animals is significant due to the risk of zoonotic transmission and the increasing use of linezolid in human healthcare to manage multidrug-resistant infections [8]. The potential for cross-selection of resistance due to the use of antimicrobials considered less important in human and veterinary medicine (such as phenicols) should be considered when developing future strategies for improved antimicrobial stewardship [47,48].

In this study, acquired resistance to quinupristin–dalfopristin among MRSA isolates was observed in 13.9% of isolates from dogs and 17.9% from cats (five isolates each). Among MRSP isolates, the acquired resistance was 12.8% in dogs and in one of the four cats. Similar results were found in a study from Spain, where 9.5% of *S. pseudintermedius* isolates from dogs were resistant to quinupristin–dalfopristin, while no resistance was observed in *S. aureus* isolates from cats [49].

To our knowledge, the acquired resistance to rifampicin observed in this study—detected in MRSA isolates (one dog, one cat), and in five MRSP isolates (four dogs and one cat)—constitutes the first documented occurrence of such resistance in Belgium. While rifampicin resistance in MRSA isolates from dogs and cats remains rare, it has been reported in other regions, including Europe and North America [8,50]. Similarly, rifampicin-resistant MRSP isolates have been reported in dogs in Italy, the Netherlands, and the USA [51,52,53] as well as in both dogs and cats in Germany [54]. Rifampicin is typically prescribed in human medicine but is also used under the cascade system in veterinary medicine, particularly for the treatment of *Rhodococcus equi* infections in foals [8]. A study from the Netherlands investigated the mechanisms of rifampicin resistance in MRSP isolated from dogs and suggested that this resistance could result from prior undocumented rifampicin use or a spontaneous *rpoB* mutation, which is known to confer rifampicin resistance [50]. As for the vancomycin-resistant MRSP (VR-MRSP) isolated from a dog, this is once again a first in Belgium, to our knowledge. VR-MRSP has been reported at low levels in the USA [53]. Additionally, a large-scale study in Scotland detected VR-MRSP in dogs, cats, and even otters. This investigation, which included isolates from both companion animals and humans, highlighted the presence of multidrug-resistant strains, although vancomycin resistance itself remains a rare finding [55]. While vancomycin use in veterinary medicine is uncommon, indirect exposure or environmental factors could contribute to the development of resistance, as suggested by broader patterns of antimicrobial resistance in *S. pseudintermedius* [55]. For these reasons, it is also relevant to introduce the Access, Watch, Reserve (AWaRe) antibiotic classification developed by the World Health Organisation (WHO) in 2017. This list includes 250 antibiotics divided into three groups: Access, Watch, and Reserve [56]. The use of both the AMEG and AWaRe classifications can be helpful in guiding rational decisions regarding the use of the cascade [51]. The results of this study showed that the highest percentages of acquired resistance are found in antibiotic classes C and D (Caution and Prudence) according to the AMEG classification, while the lowest percentages are observed in category A (Avoid).

## 4. Materials and Methods

### 4.1. Strain Collection

All samples were taken by veterinarians from clinical cases in dogs and cats during diagnostic consultations between January 2022 and January 2024. Isolates identified as presumptive MRSA or MRSP by routine diagnostics in veterinary laboratories (ANTECH and Medvet) were sent to the Veterinary Bacteriology Service at Sciensano (Brussels, Belgium) for further antimicrobial resistance characterization. Data regarding the sampled animals—except for species—and the sampling sites were missing. These presumptive identifications were based on MALDI-TOF and susceptibility testing but had not been confirmed by triplex real-time PCR (RT-PCR) targeting the *mecA* and *mecC* genes. In total, 79 presumptive MRSA isolates were collected—34 from cats and 45 from dogs—and 110 presumptive MRSP isolates—105 from dogs and 5 from cats. Only one isolate per animal was included in the study. These isolates came from 135 veterinary clinics (first line, second line, and university clinics) across Belgium, with 85 located in Wallonia and 50 in Flanders. All isolates were further analysed by RT-PCR followed by susceptibility testing.

### 4.2. DNA Extraction

The DNA samples were prepared for bacteria grown on Columbia Sheep Blood Agar plates (Biorad, Lokeren, Belgium) by using Qiagen DNeasy blood and tissue kit (Qiagen, Hilden, Germany) according to the manufacturer’s instructions.

### 4.3. Real-Time PCR

The isolates received from different laboratories were stored frozen in 1 mL of brain–heart infusion (Biorad, Lokeren, Belgium) and 1 mL of glycerol in a cryotube at −80 °C until analysis. The strains were subcultured on Columbia sheep blood agar and incubated at 37 °C for 24 h. All isolates were analysed by triplex real-time polymerase chain reaction, detecting the *Staphylococcus aureus* specific *nuc* gene and the presence of the *mecA* and the *mecC* gene variants [57]. The *nuc* gene codes for a highly conserved thermonuclease in *S. aureus* and was therefore targeted as an internal control to confirm the *S. aureus* identification. This RT-PCR, initially designed to detect *mec* genes in MRSA, has also been used for the same purpose in MRSP. The reference strains used as positive controls were MRSA ATCC 33592 (*mecA*), MRSA 2013/EURL-ST 7.1 (*mecC*), and MSSA ATCC 25293 (*nuc*). The RT-PCR reaction was carried out in a reaction volume of 25 µL consisting of 1× Roche mix (Roche, Almere, Nederland), 0.5 µM primers, 0.2 µM dual-labelled probes, and 1 µL of DNA extract [57]. Amplification consisted of Taq polymerase activation at 95 °C for 10 min followed by 40 cycles of 30 s denaturation at 95 °C, 30 s annealing at 58 °C and 30 s elongation at 72 °C.

### 4.4. Antimicrobial Susceptibility Testing

The broth micro-dilution method was used to determine the minimum inhibitory concentration (MIC) of a panel of molecules selected for their relevance in human medicine or their frequent use in veterinary medicine. The isolates were first inoculated on Columbia sheep blood agar plates and incubated at 37 °C for 24 h. Three to five colonies from these plates were added to 10 mL of sterile saline solution until a 0.5 McFarland suspension was obtained. A total of 30 µL of this suspension was inoculated into an 11 mL tube of Mueller–Hinton medium with 2-[[1.3-dihydroxy-2-(hydroxymethyl)propan-2-yl]amino]ethanesulfonic acid) (TES) and cation adjustment (ThermoFisher Scientific, Waltham, MA, USA). Then, 50 µL of this inoculum was added to each well of a Sensititre^®^ EUST2 plate (Sensititre^TM^, Thermo Fisher Scientific, Waltham, MA, USA) [58], using the Automated Inoculation Delivery System, and the plate was incubated at 35 °C for precisely 24 h. For each run, MRSA ATCC 29213 isolate was also incubated to serve as external quality control. The plates were read using a Thermo Scientific™ Vizion™ instrument and the Sensititre™ SWIN™ Software System Version 3.4 (Thermo Fisher Scientific, Waltham, MA, USA). To determine acquired resistance, the EUCAST website was consulted [28] and the epidemiological cut-off values (ECOFFs) of *S. aureus* and *S. pseudintermedius* were used, and when ECOFFs were not available, particularly for MRSP, they were approximated based on *S. aureus* values (Table 1). ECOFFs are not available for sulfamethoxazole. Therefore, the interpretative threshold for sulfamethoxazole provided in the EU Decision 2023/1017 for MRSA monitoring was applied as an alternative for both MRSA and MRSP [59]. The levels of antimicrobial resistance are described using specific terms based on the percentage of resistant isolates: “rare: <0.1%”, “very low: 0.1% to 1.0%”, “low: >1.0% to 10.0%”, “moderate: >10.0% to 20.0%”, “high: >20.0% to 50.0%”, “very high: >50.0% to 70.0%”, and “extremely high: >70.0%” [60]. Isolates with an MIC strictly higher than the EUCAST ECOFFs were referred to as ‘non-wild-type’ isolates or as isolates with phenotypically detectable acquired resistance. MDROs were designated as such when they showed acquired resistance to at least one agent belonging to at least three different antimicrobial classes [25,26]. The beta-lactams (penicillin and cefoxitin) were considered as one antimicrobial class for the MDRO assessment. Likewise, gentamicin, kanamycin, and streptomycin belong to the aminoglycoside class.

### 4.5. Statistical Analysis

The lower and upper 95% confidence limits of the sample proportions of resistant isolates were calculated per antimicrobial using the Epitools epidemiological calculators [61], based on the Wilson Confidence interval method for greater accuracy with binomial data. When sample size permitted, differences in acquired resistance proportions between dogs and cats were assessed using chi-square tests and corresponding *p*-values, also computed with Epitools [61].

## 5. Conclusions

The detection of MRSA strains carrying the *mecC* gene underscores the need to include this gene in routine RT-PCR assays, in order to refine the genetic characterization of isolates from both clinical cases and healthy animals. In parallel, improved centralization of clinical data samples submitted to laboratories would enhance our understanding of the resistance patterns observed. While this study offers valuable insights into antimicrobial resistance patterns in MRSA and MRSP strains from clinical cases in dogs and cats, the non-randomised sampling based on pre-selected diagnostic isolates means the findings reflect only the resistance profiles of confirmed cases. As such, they do not provide information on the prevalence of MRSA or MRSP infection, colonization, or carriage in the general canine and feline population in Belgium. Despite limitations in available data on sampled animals—such as sampling sites and prior treatments—the study revealed that most MRSA and MRSP clinical isolates from dogs and cats were multidrug-resistant, with some displaying resistance to antimicrobials critically important for human medicine.

Given the zoonotic potential of these pathogens and their relevance to both human and animal health, monitoring their resistance to both veterinary and human antimicrobials is strongly recommended. Such monitoring would support more informed antimicrobial therapy decisions, reduce reliance on broad-spectrum antibiotics, and help limit the further development of resistance. Integrating targeted antimicrobial use is essential to preserve the effectiveness of current treatments and ensure better health outcomes for both humans and animals.

## Figures and Tables

**Table 1:** Panel of the antimicrobial substances tested and the concentration ranges of the EUST2 plate and ECOFFs (2025) used for MRSA and MRSP.

Antimicrobial	Range Tested (µg/mL)	ECOFF MRSA (µg/mL)	ECOFF MRSP (µg/mL)
Chloramphenicol	4–64	16 *	16 *
Ciprofloxacin	0.25–8	2 *	2 *
Clindamycin	0.12–4	0.25 *	0.25 ^#^
Erythromycin	0.25–8	1 *	0.5 ^#^
Cefoxitin	0.5–16	4 *	4 *
Fusidic acid	0.25–4	0.5 *	0.5 *
Gentamicin	0.5–16	2 *	0.25 ^#^
Kanamycin	4–32	(8) *	(8) *
Linezolid	1–8	4 *	4 *
Penicillin	0.06–1	0.125 *	0.03 ^#^
Rifampicin	0.015–0.5	0.03 *	0.03 *
Sulfamethoxazole	64–512	(128) ^§^	(128) ^§^
Streptomycin	4–32	16 *	16 *
Quinupristin–Dalfopristin	0.5–4	1 *	1 *
Tetracycline	0.5–16	1 *	1 ^#^
Tiamulin	0.5–4	(2) *	2 *
Trimethoprim	1–16	2 *	2 *
Vancomycin	1–8	2 *	2 *
Mupirocin	0.5–256	1 *	1 *

* ECOFFs *S. aureus* (EUCAST 2025 [28]). ^#^ ECOFFs *S. pseudintermedius* (EUCAST 2025). ( ) tentative ECOFFs. ^§^ the interpretative threshold recommended for sulfamethoxazole for MRSA monitoring according to the EU Decision 2023/1017 was used as an alternative for both MRSA and MRSP because ECOFFs were not available.

**Table 2:** Distribution of MIC values for 64 MRSA isolates from dogs (n = 36) and cats (n = 28).

Antimicrobial Agent	Number of Strains with MIC (mg/L)														Wild-Type		Non-Wild-Type	
	≤0.03	0.06	0.12	0.25	0.5	1	2	4	8	16	32	64	128	>128	n	%	n	%
**CHL**									50	9		5			59	92.2%	5	7.8%
**CIP**				9	14	1		1	39						24	37.5%	40	62.5%
**CLI**			38	1				25							39	60.9%	25	39.1%
**ERY**				26	10			1	27						36	56.3%	28	43.8%
**FOX**									14	50					0	0.0%	64	100.0%
**FUS**				54	4	2	1	3							58	90.6%	6	9.4%
**GEN**					36				6	22					36	56.3%	28	43.8%
**KAN**								24	4	1	35				28	43.8%	36	56.3%
**LZD**						4	59	1							64	100.0%	0	0.0%
**MUP**					64										64	100.0%	0	0.0%
**PEN**						64									0	0.0%	64	100.0%
**RIF**	62				2										62	96.9%	2	3.1%
**SMX**												55	5	4	60	93.8%	4	6.3%
**STR**								32	15	9	8				56	87.5%	8	12.5%
**SYN**					43	11	10								54	84.4%	10	15.6%
**TET**					31					33					31	48.4%	33	51.6%
**TIA**					46	12		6							58	90.6%	6	9.4%
**TMP**						24	5			35					29	45.3%	35	54.7%
**VAN**						63	1								64	100.0%	0	0.0%

The line represents the ECOFFs (see Table 1), showing the separation between the wild-type strains and the non-wild-type strains. FOX: Cefoxitin; CHL: Chloramphenicol; CIP: Ciprofloxacin; CLI: Clindamycin; ERY: Erythromycin; FUS: Fusidic acid; GEN: Gentamicin; KAN: Kanamycin; LZD: Linezolid; PEN: Penicillin; RIF: Rifampicin; STR: Streptomycin; SMX: Sulfamethoxazole; SYN: Quinupristin–Dalfopristin; TET: Tetracycline; TIA: Tiamulin; TMP: Trimethoprim; VAN: Vancomycin; MUP: Mupirocin.

**Table 3:** Distribution of MIC values for 106 MRSP isolates from dogs (n = 102) and cats (n = 4).

Antimicrobial Agent	Number of Strains with MIC (mg/L)														Wild-Type		Non-Wild-Type	
	≤0.03	0.06	0.12	0.25	0.5	1	2	4	8	16	32	64	128	>128	n	%	n	%
**CHL**								21	64	2		19			87	82.1%	19	17.9%
**CIP**				18	3	1			84						22	20.8%	84	79.2%
**CLI**			8	2			1	95							10	9.4%	96	90.6%
**ERY**				8					98						8	7.5%	98	92.5%
**FOX**					6	18	16	12	40	14					52	49.1%	54	50.9%
**FUS**				92	2		1	11							94	88.7%	12	11.3%
**GEN**					10			6	27	63					0	0.0%	106	100.0%
**KAN**								4			102				4	3.8%	102	96.2%
**LZD**						105			1						105	99.1%	1	0.9%
**MUP**					103	3									106	100.0%	0	0.0%
**PEN**					1	105									0	0.0%	106	100.0%
**RIF**	101				5										101	95.3%	5	4.7%
**SMX**												14	12	80	26	24.5%	80	75.5%
**STR**								8	1		97				9	8.5%	97	91.5%
**SYN**					78	13	12	3							91	85.8%	15	14.2%
**TET**					10			2		94					10	9.4%	96	90.6%
**TIA**					92		1	13							93	87.7%	13	12.3%
**TMP**						4	6	2		94					10	9.4%	96	90.6%
**VAN**						105		1							105	99.1%	1	0.9%

The line represents the ECOFFs (see Table 1) showing the separation between the wild-type strains and the non-wild-type strains. FOX: Cefoxitin; CHL: Chloramphenicol; CIP: Ciprofloxacin; CLI: Clindamycin; ERY: Erythromycin; FUS: Fusidic acid; GEN: Gentamicin; KAN: Kanamycin; LZD: Linezolid; PEN: Penicillin; RIF: Rifampicin; STR: Streptomycin; SMX: Sulfamethoxazole; SYN: Quinupristin–Dalfopristin; TET: Tetracycline; TIA: Tiamulin; TMP: Trimethoprim; VAN: Vancomycin; MUP: Mupirocin.

**Table 4:** Resistance profiles of the MRSP isolates from 4 cats according to the number of antimicrobial classes from which they acquired resistance.

Sample Reference	Resistance Profiles	Number of Antimicrobial Families
CT 66	CIP-ERY-GEN-KAN-PEN-TMP	5
CT 71	CIP-CLI-ERY-FOX-GEN-KAN-PEN-SMX-STR-TET-TMP	8
CT 85	CIP-CLI-ERY-FOX-GEN-KAN-PEN-RIF-SMX-STR-TET-TMP	9
CT 58	CIP-CLI-ERY-GEN-KAN-LZD-PEN-SMX-STR-SYN-TET-TIA-TMP	11

FOX: Cefoxitin; CHL: Chloramphenicol; CIP: Ciprofloxacin; CLI: Clindamycin; ERY: Erythromycin; FUS: Fusidic acid; GEN: Gentamicin; KAN: Kanamycin; LZD: Linezolid; PEN: Penicillin; RIF: Rifampicin; STR: Streptomycin; SMX: Sulfamethoxazole; SYN: Quinupristin–Dalfopristin; TET: Tetracycline; TIA: Tiamulin; TMP: Trimethoprim; VAN: Vancomycin; MUP: Mupirocin. Sensititre^®^ EUST2 panel.

## Data Availability

The raw data supporting the conclusions of this article will be made available by the authors on request.

## Supplementary Materials

The following supporting information can be downloaded at: https://www.mdpi.com/article/10.3390/antibiotics14070631/s1, Table S1: Resistance profiles of the 64 MRSA isolates from dogs (n = 36) and cats (n = 28). Table S2: Resistance profiles of the 106 MRSP isolates from dogs (n = 102) and cats (n = 4).
